# Treatment of Scarlatina by Diluted Acetic Acid

**Published:** 1857-08

**Authors:** B. F. Schneck

**Affiliations:** Lebanon, Pa.


### DEPARTMENT OF SELECTIONS.

### Treatment of Scarlatina by Diluted Acetic Acid.

BY B. F. SCHNECK, M. D., LEBANON, PA.

During the past twelve or fourteen months a severe epidemic of scarlatina has prevailed in my neighborhood. Of 190 cases of the disease treated by me in accordance with the method recommended by our best authorities, I lost 1 in $8\frac{1}{2}$ to 9.

Disatisfied with this result, I was induced to try the diluted acetic acid as recommended by Dr. I. B. Brown, whose work* I had the good fortune to meet with at the commencement of the present year. Of 60 cases treated subsequently by this plan, I did not lose one. The disease at this time had not undergone any abatement from its former violence; for among the sixty recoveries there were cases of such malignancy, as would inevitably have perished under the best directed previous efforts. It is true that two out of the sixty died afterwards of thoracic and cerebral dropsy; and one, after nearly a two weeks' convalescence, from purpura hæmorrhagica, with epistaxis, hæmaturia, &c.; but these cases cannot be regarded as affecting the integrity of the plan in question. I am thus enabled to bear a flattering testimony to the success of Dr. B.'s method.

Many medical men, after unsatisfactory trials of all the ordinary modes of treatment, now declare that the less there is done for scarlatina the better. All such will be apt to think lightly of Dr. Brown's method; if, indeed, they do not condemn what they may choose to call his *nimia cura medici.* Let such rest assured, however, that this is a disease, which, like weeds, flourishes most when least attended to; and further, that the character of medical adviser must be merged, for the time, in that of nurse also, to a certain extent, if his ministrations are to be successful. He should see his patients several times a day—the oftener the better—and, following the example of our author, he should even be found holding nightly vigils

* On the Treatment of Scarlatina by the Acidum Aceticum Dilutum of the Pharmacopœia. By I. B. Brown, M. D., London, 1846.

by the bedside, if the urgency of the case required it. The daily dressings of the fauces with caustic should, if possible, be made by himself; he should direct the frequency of the repetition of stimulants; and even the minutest details should ever be under his immediate cognizance. Thus fully occupied, although he may be able to take charge of fewer patients, he will save more lives; and only thus will he be able to realize the truth of the otherwise almost incredible statement of a friend of the author's, engaged in extensive practice, who writes that "the number of fatal cases occurring to him under this treatment did not exceed *four.*" This gratifying result, it is the writer's firm conviction, will be the reward of all who will adopt and faithfully carry out the plan.

The following is a synopsis of Dr. Brown's views:

1. Scarlatina is always and essentially a disease of, or tending to debility, and not of an inflammatory nature. Its poison acts primarily and most fatally upon the blood, producing a dissolved, semi-vitalized and putrescible condition of that fluid; so that it possesses more serum and less fibrin than in its normal state. "Consequently the serum percolates, or is effused into the cellular tissue and cavities, through the coats of the vessels. Salines favor this dissolved state of the blood; but acetic acid prevents the separation of the serum from the fibrin."

2. Acetic acid is an excellent antiseptic; "it gives tone to the blood in scarlatina, and prevents the separation of the serum from the fibrin." It also acts as an astringent upon the lymphatic system and serous membranes, and so effectually prevents dropsy."

3. It is a grateful refrigerant.

4. No midicine has a more decided influence in promoting digestion than this acid. We are further directed, while administering it, to "allow patients almost anything they fancy; it will seldom hurt them in severe and even dangerous disease."

These four points lie at the foundation of Dr. Brown's very simple and very successful treatment. The specialities of his method will now be given, as applicable to the several forms of the disease.

Whatever may be the type, he prepares the system for the acid, by giving—

1. *An aperient* of 3 to 5 grs. of calomel, to be followed in two hours by castor oil. All *saline* aperients are condemned; "salines favor a dissolved state of the blood." If, from great gastric irritability, the oil is rejected, he recommends an aperient mixture (rhubarb and magnesia,) which contains no saline substance.

2. Apply a piece of flannel round the throat from ear to ear, saturated with soap lin. f oz j; camphor lin., laud. aa drach ij.—M.

3. After the operation of the oil, give—for a patient nine years old—distilled vinegar, diluted,* f oz j; syrup f drach iv; distilled water f oz iv.—M. Two tablespoonfuls every four hours. This mixture is to be continued throughout the entire duration of the case, whatever the form of the disease; and for one or two weeks afterwards, or until desquamation is well over. "It acts as an astringent upon the lymphatic system and serous membranes, and so effectually prevents dropsy."

4. Whenever, in scarlatina simplex, there is slight delirium in the beginning, with a thick, viscid phlegm on the the tonsils, apply daily—nitr. silver grs. x; distilled water f oz j.—M. You thus prevent s. anginosa. If the throat require it, a linseed poultice may be placed over the flannel, and kept there constantly.

5. On the third or fourth day, in simple cases, allow mutton-broth.

6. As soon as desquamation comes on, order a warm bath or two, and keep the patient strictly in bed during the whole process.

*S. Anginosa.*—Here the treatment is the same, except that the caustic must be used more frequently, and the proportion of acid in the solution must be increased. A good rule is to increase the strength according to the violence of the attack, in bad cases giving it as strong as the patient can take it. Poultices to throat. Should symptoms of adynamia come on, give arrowroot, with a spoonful of brandy in it; add comp. sp. ether to acid solution; wash face, hands, legs, and chest with tepid vinegar ($\frac{1}{3}$) and water ($\frac{2}{3}$). If restless at night, give tinct. hyoscyam., or $\frac{1}{8}$ to $\frac{1}{4}$ gr. morphia, according to age. The decoction of bark may also be added to the acid mixture. Whenever, in s. anginosa, symptoms of adynamia come on, dress the throat frequently with caustic, and increase the quantity of acid from day to day; you thus prevent s. maligna.

For adults, in cases partaking of the nature of s. maligna, the following formula is given: ℞.—Distilled vinegar f drach iv; syr. red poppies f drach. iv; distilled water f oz iv.—M. One-fourth part to be taken every four hours.

In s. maglina the same course of treatment is to be pursued; calomel, oil, caustic, acid mixture (strong,) liniment or sinapism to throat, followed by poultices; brandy or port wine every four to six hours, with arrowroot, beef-tea, or mutton-broth;

---

*℞.—Distilled vinegar, offic., one part; water seven parts.—M.

morphia at bed time, or whenever restless, and sponging with tepid vinegar and water. All the bed furniture, carpets, &c., to be removed from the room, and chloride of lime to be sprinkled about the floor. During desquamation the patient is not to sit up at all. Give at this time one or more warm baths; use wine and brandy in s. maligna, even in the febrile stage; when combined with the acid, which so powerfully assists digestion, no harm will ever accrue from their use.

This is a brief statement of Dr. Brown's views and practice in this terrible malady. To the work itself we must refer for a number of valuable cases, illustrating most happily the treatment which he advocates, and interspersed with many highly practical remarks. I cannot help transcribing at length, as a fitting close to this portion of the subject, the following observations, which I have copied from his work:—

"Very much depends on careful watching in this disease; there is always, in one or the other of the stages, a *critical moment.* For instance, in the eruptive stage, even in s. simplex, delirium will come on, and the throat will become more clogged with viscid secretion in a few hours; and if attention be not promptly given, and this phlegm, which impedes free respiration, be not removed, the delirium and laborious breathing will increase, and the disease will soon run into the second or anginose form. In this case, the throat must be promptly cleansed, and some gentle nourishment be given. Again, in s. anginosa, it will not seldom happen that the tonsils and fauces will suddenly become worse, or great sickness or sudden prostration will come on; now, unless the throat be instantly attended to, delirium, laborious breathing, difficult deglutition, and restlessness will make serious ravages upon the patient, and all remedies will quickly become unavailing; or, where sudden prostration should arise, then we must promptly and unsparingly administer stimulants and cordials till the pulse exhibits more steadiness and power."

The practical importance of these directions cannot be overestimated. As assisting the cleansing of the fauces from viscid secretions, I have, for several years past, been in the habit of injecting the diluted chlorinated soda into the nares, with the happiest effects. Extensive ulceration, not only of the posterior nares, but of the entire nasal tract, with an abundant secretion of a peculiar tenacious mucus, are an attendant on every bad case; and these passages cannot be long obstructed without great distress and imminent danger. The daily or bi-daily injection of Labarraque's solution, therefore, while it effectually clears away the obstruction (as any other liquid would as well), exerts besides an alterative and healing influence upon the ulcerated surface itself; and it *destroys*, while it removes, the morbid products which, if swallowed, as they are otherwise sure to be, disturb so seriously the intestinal canal; and last, but not least, it corrects the fetor which is so disagreeable a concomitant of such cases. So signal is the relief derived from this procedure, that, unpleasant though the sensation must be, I have seen the little patients, instead of shrinking from the operation, instinctively court the repetition of it, and if old enough, ask for it. It is a measure which, in the class of cases referred to, cannot be dispensed with, without loss. But as it may happen that a considerable quantity of the injection may be swallowed, and the blood be thereby impaired, it will be proper always to precede or follow the injection with a strong dose of acetic acid, so as to neutralize the saline ingredient.

The preparation of the acetic acid solution may be varied somewhat from the formulas given above, and so simplified, without in the least affecting the result. Instead of first diluting the concentrated acid to the strength of vinegar, then using the dilution for the preparation of the solution, I have been accustomed merely to add from f drach. j to f drach. iv of the officinal acid to f oz. iv water, and ordering a tablespoonful every few hours, sweetening at the time of administering it. We must, however, never forget to increase the strength in proportion to the threatening nature of the symptoms.

In the use of stimulants, also, a little license has been taken with our author's directions. Having ventured upon the guarded employment of brandy, beef-essence, &c., as a precautionary step, earlier in the attack than he allows, without detriment, I now administer brandy in graduated doses, two or three times a day *from the beginning* in the malignant form, or on the second or third day in anginose cases; and I have seen no reason to regret this course. If the tongue becomes red like a strawberry, with the papillæ as large as a pin's head, or on the contrary, brown, dry, fissured, with sordes on the teeth; and if there be, besides, a recession of the eruption, a pulse fluttering and not to be counted, or even delirium, "then we must unsparingly administer stimulants and cordials, until the pulse exhibits more steadiness and power." Carb. ammonia, quinia, and even capsicum, have here all failed me; this last having proved alike ineffectual as an arterial stimulant, and as a local application to the fauces.

If Scarlatina were an inflammatory disease, as the advocates of bleeding and antiphlogistics would have us to believe, such a stimulant course could not fail to result disastrously in nearly every instance; but the reverse is actually the fact. The violent excitement in severe attacks, as indicated by burning skin, rapid pulse, dilirium, &c., is not an evidence of phlogosis, but of irritation. And when death takes place in such cases, it is not so much from inflammatory disorganization of any vital part, as from shear exhaustion; the inevitable consequence of the excitement into which the system had worked itself, in its vain struggles against the fatal poison which was oppressing it.

Dr. Brown's silence in regard to the use of emetics is a significant fact; although more celebrated authorities than he, recommend them highly. Their adoption at all, as part of the treatment, was probably suggested by the nausea and vomiting which almost always usher in the attack; under the supposition of the presence of acrid ingesta, which they were designed to remove. It may be, that when the mildest article is selected, solely with this view, they may do no harm; but when administered indiscriminately, fatal results must occasionally follow the practice.

Dentition, improper food, the hot months, and hereditary predisposition, may all, in scarlatina, favour the occurence of serious gastro-intestinal disease, from the least exciting cause; and an emetic, especially if containing tart. antimony as advised by some may be this cause. In the month of July, 1856, I was called to see a child aged 20 months, ill with s. anginosa, running into maligna, with scarcely any eruption. Notwithstanding the child had vomited, an emetic of ipecacuanha with calomel was given, after a warm bath; to be followed by sp. nitric ether and bicarb. soda in solution, with capsicum infusion. The vomiting became unmanageable, attended with copious diarrhœa; gastritis supervened, with peritonitis and enormous abdominal distention; and on the fourth day the child died in convulsions. The emetic most probably had killed it.

What, let us ask, does the gastric irritability of this disease mean? Is it not the first appreciable alarm given by nature of the introduction of the poison, and an ineffectual attempt on the part of the system, to get rid of it at the outset? But as the morbific matter is introduced, and the blood saturated with it, many days it may be before it actually develops itself, how can we expect emesis, whether spontaneous or artificial, to dislodge it? If, instead of vomiting, scarlatina began with diarrhœa, would we be justified in giving an active purgative, with the same object? Assuming Dr. Brown's view to be correct, would it not be malpractice to bring to bear the depressing effects of a nauseating emetic upon a disease whose tendency from the beginning is towards debility? The unfortunate result above related has convinced me that the use of emetics, as a matter of routine, is fraught with great danger; and that their employment is indicated in very few, and very special cases, if at all.

The following cases, representing the worst forms of s. anginosa and maligna, are selected out of a number of similar ones, from my case-book, as illustrating the gratifying success of the acetic acid treatment, even when under the most unfavorable circumstances.

Case I.—Dec. 27, 1856. Saw a girl of Jos. Heilman, aged 13, in an attack of s. ang. threatening maligna. On the evening of the 28th found more fever, very frequent, angry pulse, constant sighing and heaving of the breath, with increased impulse of heart. Suspicion of pericarditis, and tempted to bleed. Concluded to postpone till next morning; ordering sinapisms to extremities, and dose calomel. Was prevented from seeing her until next day towards evening.

29th. Pericarditis now clear. Bled viii oz.; epispastic to left chest; cal. and op. aa $\frac{1}{4}$ gr. every two hours; sinapisms to extremities. Eruption well out. Teaspoonful brandy at one, to be continued 3 or 4 times a day, with beef-essence.

30th. Effusion around heart; impulse scarcely perceptible to hand, or audible; at times delirious; eruption well out; slight epistaxis. Inunction with mercurial oint., and same to blister. Continue remedies.

31st. Morning. Pulse more full and a shade slower; impulse of the heart more perceptible, and less muffled; had three or four evacuations. Continue treatment, with alternate doses of pulv. scillæ and digital., aa $\frac{1}{2}$ gr., cal. $\frac{1}{4}$ gr.

Evening. Cardiac trouble decidedly better; but alarming prostration, from epistaxis to the extent of a pint. Partial coma; tongue dry and papillæ very much elevated; four alvine discharges; Cold cloths to head and neck; Dover's p., 3 grs., digital. $\frac{1}{2}$ gr. acet. lead, $\frac{1}{2}$ gr. every 2 hours, (having omitted former powders;) 10 drops elix. vitriol every 2 hours. Sinapisms to extremities; iced lemonade, drink; may die to-night.

Jan. 1, 1857. Morning. Bled a pint or more at two several times to-night; extremely exhausted; but one dose of the medicines ordered last evening was given; family expected her death hourly. This being contrary to my express orders, I at once directed a resumption of the treatment, including brandy and essence of beef.

Evening. Has taken remedies all day; no bleeding. Pulse a little fuller, and slightly slower. Tongue dry and covered with crusts of blood. Eruption apparently about to decline on upper part of the body, but well out on lower extremities. Continue treatment, at three hours' interval. 2nd—Noon. Pulse a little slower; circumscribed flush on each cheek; face sunken; tongue very dry; skin dusky, and whole case *typhoid*. Turpentine, emulsion and elix. vitriol, with beef-essence, and brandy and milk.

3d. Tongue a little more moist. Continue remedies.
4th. Improving; pulse a little slower. Will recover.
5th to 6th. Has great appetite. Slowly convalescent.

*Remarks.*—Bleeding, in scarlet fever, is not necessarily an injurious measure, especially if its otherwise depressing effect be guarded against, immediately afterwards, by suitable doses of stimulants and nourishment. In this instance, the venesection most assuredly saved life, by moderating and favoring the resolution of cardiac inflammation; which, although it had gone on to the effusion of serum, was nevertheless relieved by it, and by the subsequent use of squill, digitalis and calomel. The recession of the eruption, which might otherwise have followed the bleeding, was also prevented by the prompt administration of small doses of brandy. In a similar case of pericarditis in the course of scarlatina, I should feel emboldened to bleed largely, giving stimulants and beef-tea generously immediately afterwards, as the only mode promising success.

CASE II.—*S. Anginosa running into Maligna.*—Dec. 30, 1856. Girl of Geo. Strohm, aged 4 years. Vomiting; very rapid, irritable pulse; eruption of a vivid red color; tonsils greatly enlarged, and covered with lymphy exudations. Solid caustic to throat; cal. oil, and strong acid solution.

Jan. 1st, 1857—Morning. Symptoms of great malignancy; fauces of a dark purple hue; face mottled with white patches, where the eruption showed a disposition to recede; excessive restlessness all night, getting out of bed in the delirium; surface of an intensely deep red color; pulse rather feeble and slow. Solid caustic to throat; sinapism externally, to be followed by poultices. Teaspoonful of brandy every 5 or 6 hours, if not gone to sleep. Beef-essence; acid solution stronger.

Evening. Has slept some hours; face more uniformly red; pulse more frequent; surface hot. Sol. 10 grs. nitr. silver to oz. j water, to fauces twice a day; chlorinated soda injections into nares. Continue remedies.

2d. Same as last evening. Comp. camph. lin. to throat, which is much swollen; caustic, injections, brandy and beef-tea.

3d. Desquamation already beginning on different parts of the body, being only the fifth day—a bad sign. Continue remedies.

4th. Throat very much swollen externally; tonsils deeply ulcerated; case very malignant; sinking and very restless; surface pale and cool.

10, P. M. Was sent for; supposed to be dying. Prognosis very bad. Solid caustic to throat; injection into nares; brandy every two or three hours, and continue remedies.

5th. Pulse a shade slower. Family did not attend to throat this morning. Applied caustic at once, and injected chlor. soda into nares, bringing away large masses of viscid secretions, with great relief. Quite rational.

6th to 10th. Pulse slower. Gradually convalescent.

*Remarks.*—This case exhibited what I have repeatedly seen in this epidemic—a succession of pure white patches in the midst of the eruption, on the face most generally; appearing in the course of a few minutes, and persisting sometimes for half a day or longer. Having met with this symptom only in cases of a malignant character, with a cool skin and other signs of adynamia, I have come to regard it as an indication for the prompt use of stimulants.

The only occurrence of desquamation in this case—on the fifth day of the eruption—is also worthy of note, as indicating great depravity of system. In September, 1856, I met with a case in which desquamation began, all over the body, in extensive patches, on the fourth day of the eruption. The skin was as though it had been seethed or scalded; the cuticle separating first at the points of pressure from the motions of the patient, incident to her changes of posture in the delirium—as the elbows, hips &c.—but finally coming away wherever the clothing lay in contact with it. These denuded surfaces were literally raw; when recent, serum standing upon them in minute drops. The patient, a girl of 15 years, died rapidly of pericarditis.

CASE III.—*Purpura following S. Anginosa and Maligna.*—Feb. 23, 1857. In this instance, as in a considerable number of others in this epidemic, I observed that the eruption on the arms was most fully out along the course of the nervous trunks, there being a broad belt, of an intensely red colour, in the line of the bloodvessels and lymphatics, from the hand to the axilla. Having never seen this symptom noticed, and having observed it only in the worst forms of the disease, I have been led to regard it as indicating either phlebitis, or inflammation of the absorbents, and, as such, a serious complication of the case. The details of this case are very similar to those previously given, and hence need not be gone over. It is sufficient to say that the child recovered with the greatest difficulty; but by the end of the first week of March he was clearly convalescent, although greatly reduced, and *very pale.* He, however, took nourishment, with acid mixtures, and it was hoped he would do well.

March 13.—I was informed this morning that his mouth bled slightly, and that the blood appeared to ooze from the gums. Sent him tinct. chlorid. iron, and saw him in the afternoon. Found that epistaxis had set in; the blood looking pale red in color, like a mixture of currant-juice and water. Purpura patches had appeared over the whole of the lower extremities. Prognosis very unfavorable. Beef-essence and elix. vitriol at short intervals, alternating with sol. potassio-tartr. iron.

14th. Getting worse rapidly. Purpura on arms and breast. In the course of the day, vomiting of coagulated blood, which had evidently passed into the stomach from posterior nares. Vomiting continued; everything was rejected; and in the afternoon, after having passed some bloody urine, the child died, perfectly blanched.

*Remarks.*—This case is interesting, as confirming, to some extent, Dr. Brown's views of the pathology of scarlatina. Here was, first, a deficiency of red globules in the blood, as was evident from its pale red color. We infer, also an increased tenuity in this fluid, as manifested by the hemorrhagic tendency, and which may have been caused either by a deficiency of fibrin, or a preponderance of serum, from paucity of red corpuscles. However we may explain the morbid result, the occurrence of purpura is almost inexplicable under the constant administration of the strongest nourishment and acid solution, unless we admit the co-existence of the scarlatina poison, acting upon the blood to bring it into this dissolved state. At least, this was not congestive or inflammatory purpura.

Would it not be advisable, in every case of s. anginosa and maligna, especially the latter, to administer, as soon as the disease has subsided, and desquamation is beginning, a mild preparation of iron? Might not the fatal termination in this case perhaps have been averted by the earlier employment of a ferruginous tonic? Further, would not also the iron, by increasing the crasis of the blood, lessen the chance of dropsy? Or, on the other hand, would the iron be capable of increasing the tendency to dropsy, by rendering the blood inflammatory, and so favouring the renal disease, which is so prominent a symptom (if not the cause) of the dropsy? This is quite possible, regarding, as I do, the condition of kidney in the dropsy of scarlatina as a real, though temporary, acute Bright's disease.

Supposing, however, as does Dr. Brown, that the watery condition of the blood after scarlatina is the cause of the effusion, how can we reconcile with this the benefit derived from venesection in dropsy? If this supposition be correct, are we not, by the abstraction of blood, and the consequent still further impoverishment of that fluid, increasing the tendency to effusion? Instead of which, we find the swelling mostly soon to disappear rapidly after bloodletting. At least, such has been my experience, repeatedly, in bad cases of cerebral and cardiac dropsy; and Watson, in similar cases, gives bleeding his unqualified approval.

These facts militate strongly against the causation of dropsy, as explained by Dr. Brown. For the present, then, we know of no solution of the difficulties presented to us above, and must be content to follow apparently opposite indications, if correct and successful, without being able to reconcile differences.

Case IV.—*Scarlatina in Childbed. Scarlatina Neonati.*—On the 2d of July, 1856, I was requested to see the wife of Fred. Schaffer, in an attack of s. anginosa. She was at the end of her pregnancy, and expected her confinement daily. Both of her children had just passed through a severe attack of the disease, and she had been their only nurse. Knowing the disastrous consequences to be apprehended from scarlatina during confinement, I undertook the case with no little anxiety. On the 4th, the premonitory symptoms of labor appeared, which I treated with anodynes, hoping to put off the evil day as long as possible. Moreover, dreading the exhaustion which would be likely, in such a case, to follow the excitement of labor, and still more the debility consequent upon the lochia (which would act as a drain upon the system,) I sought to prepare the patient for the crisis by moderate doses of carbonate of ammonia, serpentaria, and beef-essence. By a cautious use of opiates, the labor was kept off until the afternoon of the 6th, when the woman was delivered of a mature female child, which, however, lived only three or four hours. This child was covered from head to foot with the eruption, of an intensely red colour; and, lest I might have mistaken the naturally florid colour of many newly-born children for scarlatina, I examined the fauces, and was surprised to find prominent anginose symptoms, and the soft palate thickly studded with red points. The infant soon became cold, and the eruption changed to a purple hue, which, before death, gave place to an almost indigo colour.

My precautions in regard to the mother proved to be well-timed. In addition to the supporting plan adopted before confinement, she now bore well a generous supply of wine. She made a good recovery; but, a week afterwards, was attacked with subacute rheumatism of the wrists, which yielded to Dover's powders and vinum colchici.

*Remarks.*—Ramsbotham, in his work on Parturition, highly recommends a stimulating aud supporting treatment of the scarlatina of puerperal women, as the only method likely to prove successful; and the above case is interesting, as confirming not only his own views, but also those of Dr. Brown. Morris, in his *Lectures on Scarlet Fever*, says that "to pregnant and puerperal women it is almost inevitably fatal. I have known several cases which proved mortal, but have never heard of a recovery."

These cases, from my own observations, must suffice for my present purpose. They confirm, and correspond with, Dr. Brown's teachings and cases very fully; and this correspondence between two epidemics thus widely separated as to time and space is certainly more than a mere coincidence. It seems to indicate a certain general principle, which underlies, and so essentially determines the nature of this, as of every other affection, through all the variations of climate, locality, and prevailing type of disease. Whether this principle, which Dr. Brown professes to have discovered as regards scarlatina, be the correct one, can only be determined after extensive and frequently repeated experiments.

Finally, to all the evidence adduced by Dr. Brown in favour of the preservative effects of acetic acid upon the blood, it is proper to oppose the testimony of our best American authority, as to its injurious effects in large and long-continued doses. Dr. Wood, in his *Therapeutics*, says that, thus administered, besides producing gastric and intestinal irritation, "it lowers the organic functions of the system generally, impairing nutrition, depraving the blood, producing anæmia and emaciation, and ultimately, it is said, inducing a condition analogous to the scorbutic." The same writer refers to its liability to develop the tubercular diathesis, when taken habitually, as it sometimes is, with a view to obviate fatness. Whether, and to what extent, Dr. Brown's use of the article should be considered toxical, it would be difficult to say; but probably the diluted state in which it is given, and the comparatively short time that it is administered, will save it from being so regarded, except in so far as many of our best remedies are poisons, in over-doses.

